# Two Distinct Bacterial Biofilm Components Trigger Metamorphosis in the Colonial Hydrozoan Hydractinia echinata

**DOI:** 10.1128/mBio.00401-21

**Published:** 2021-06-22

**Authors:** Huijuan Guo, Maja Rischer, Martin Westermann, Christine Beemelmanns

**Affiliations:** a Leibniz Institute for Natural Product Research and Infection Biology—Hans Knöll Institute, Jena, Germany; b Electron Microscopy Centre, Friedrich Schiller University Jena, Jena, Germany; Harvard University

**Keywords:** *Hydractinia*, *Pseudoalteromonas*, metamorphosis, phospholipids, polysaccharides, biofouling, exopolysaccharide, marine microbiology, microbial ecology, natural products, phospholipid-mediated signaling

## Abstract

In marine environments, the bacterially induced metamorphosis of larvae is a widespread cross-kingdom communication phenomenon that is critical for the persistence of many marine invertebrates. However, the majority of inducing bacterial signals and underlying cellular mechanisms remain enigmatic. The marine hydroid Hydractinia echinata is a well-known model system for investigating bacterially stimulated larval metamorphosis, as larvae transform into the colonial adult stage within 24 h of signal detection. Although *H. echinata* has served as a cell biological model system for decades, the identity and influence of bacterial signals on the morphogenic transition remained largely unexplored. Using a bioassay-guided analysis, we first determined that specific bacterial (lyso)phospholipids, naturally present in bacterial membranes and vesicles, elicit metamorphosis in *Hydractinia* larvae in a dose-response manner. Lysophospholipids, as single compounds or in combination (50 μM), induced metamorphosis in up to 50% of all larvae within 48 h. Using fluorescence-labeled bacterial phospholipids, we demonstrated that phospholipids are incorporated into the larval membranes, where interactions with internal signaling cascades are proposed to occur. Second, we identified two structurally distinct exopolysaccharides of bacterial biofilms, the new Rha-Man polysaccharide from *Pseudoalteromonas* sp. strain P1-9 and curdlan from Alcaligenes faecalis, to induce metamorphosis in up to 75% of tested larvae. We also found that combinations of (lyso)phospholipids and curdlan induced transformation within 24 h, thereby exceeding the morphogenic activity observed for single compounds and bacterial biofilms. Our results demonstrate that two structurally distinct, bacterium-derived metabolites converge to induce high transformation rates of *Hydractinia* larvae and thus may help ensure optimal habitat selection.

## INTRODUCTION

The radical transformation (metamorphosis) of motile larvae into the adult stage is a critical step in the life cycle of many marine species, as it allows the propagation and persistence of the population in a marine ecosystem (1). It has been now recognized for over 80 years that chemical signals present within marine biofilms induce settlement and metamorphosis in larvae (2–4). However, the identification of these signaling molecules remains a challenging task, and only a few key bacterial signals have been structurally characterized and functionally analyzed (5–7). One of the few identified bacterial signaling molecules is bromopyrrole, produced by *Pseudoalteromonas*, which induces metamorphosis in coral larvae (8, 9). However, it has been found that larvae fail to attach to surfaces when stimulated by bromopyrroles alone, and it was deduced that other, still unidentified, chemical cues are key to the morphogenic process.

Recent biochemical investigations of the bacterium-induced metamorphosis of the marine polychaete Hydroides elegans resulted in the identification of phage tail-like contractile injection systems (tailocins) in *Pseudoalteromonas* species. These injection systems induce settlement and metamorphosis by releasing an effector protein Mif1, which stimulates the p38 and mitogen-activated protein kinase (MAPK) signaling pathways (10–12). However, bacteria not capable of producing these proteinaceous injection systems were also found to induce the transformation, presumably by other, not-yet-identified morphogens (13, 14). In the 1990s, Leitz and Wagner reported that a lipid-like molecule of Pseudoalteromonas espejiana (formerly Alteromonas espejiana) induces larva transformation in Hydractinia echinata, an early-branching metazoan lineage dating back more than 500 million years (15). Despite intensive studies, however, the bacterial signals causing *Hydractinia* larvae to metamorphose have remained elusive. As such, it has been typical to artificially induce metamorphosis of *Hydractinia* using high salt concentrations (CsCl). Although this method is effective, it also causes phenotypical and initial developmental differences during the morphogenic process compared to bacterial induction (16, 17).

To close this knowledge gap and yield new insights into the natural bacterium-host interaction, we investigated which bacterial species and metabolites are naturally abundant in marine biofilms that cause *H. echinata* to settle and metamorphose. Thus, we set out to characterize the bacterial morphogens from associated *Pseudoalteromonas* species and phylogenetically related bacterial lineages using a bioassay-guided isolation approach. Here, we report on identification and structure-activity studies of two types of bacterial metabolites that, both alone and in combination, stimulated *H. echinata* larvae to metamorphose into the adult stage. Our results also suggest that outer membrane vesicles (OMVs) and the extracellular matrix of biofilms play essential roles in the prokaryote-eukaryote signaling mechanisms in this organism and likely also those of many other benthic marine invertebrate animals.

## RESULTS

### Bioassay-guided identification of morphogenic bacteria.

**FIG 1:**
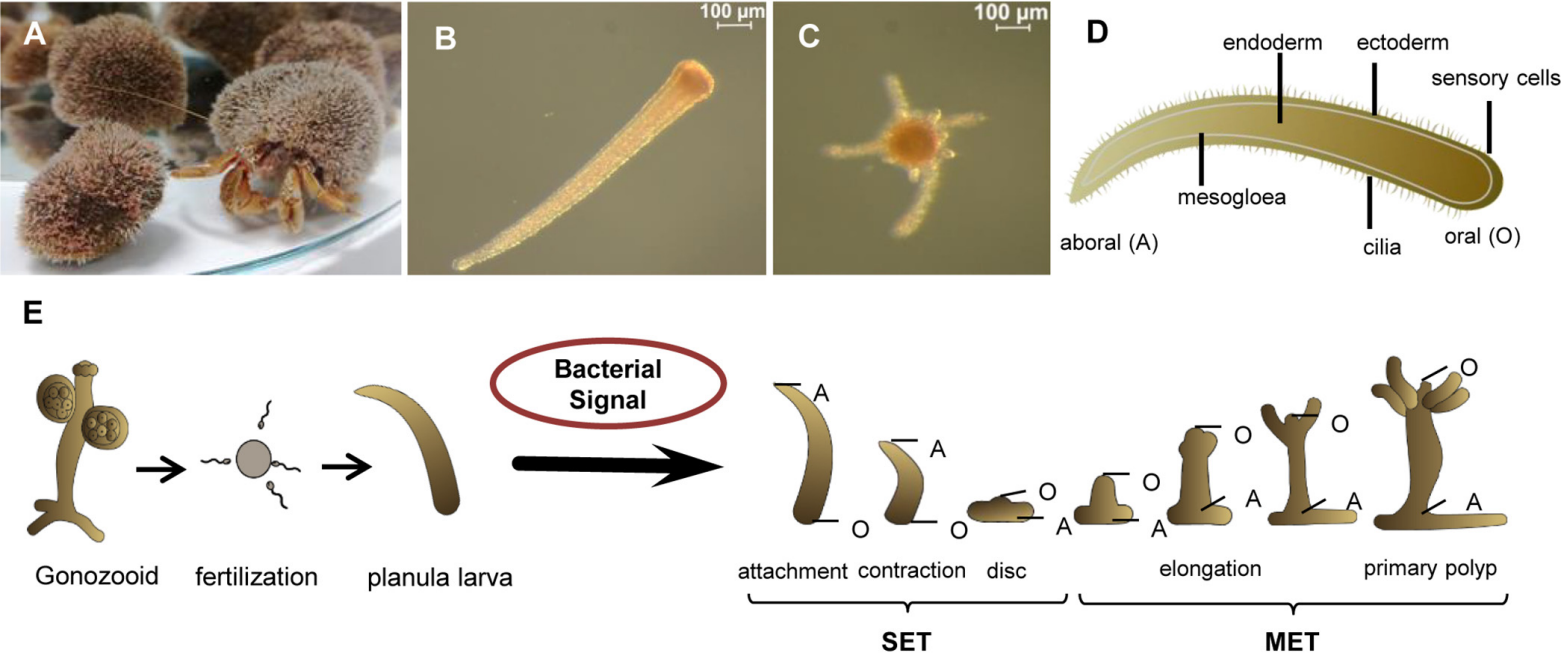
(A) *Hydractinia echinata* colonizing the shell inhabited by a hermit crab (*Pagurus* sp.). (B and C) Microscopic images of *H. echinata* planula larva (B) and primary polyp (C). (D) Sketch of a larva. (E) Generalized sketch of the life cycle of *Hydractinia echinata* showing the different stages of the transformation process of induced larvae (SET = transformation process until disc formation; MET = transformation into a fully functional primary polyp).

Fertile *H. echinata* polyps release their eggs or sperm in a light-controlled process, after which the eggs are fertilized. Within 72 h, an embryo passes through several developmental stages into a motile larva that immediately starts exploring benthic surfaces. Upon detection of signals derived from marine biofilms, a larva metamorphoses into a primary polyp within 24 to 48 h, which propagates to become the adult colony. Although early reports indicated that bacterial isolates of different lineages (e.g., *Pseudoalteromonas* spp., *Oceanospirillum* spp., Staphylococcus aureus, Pseudomonas spp., and Serratia marinorubra) and derived from marine biofilms are capable of inducing metamorphosis (18), many of the earlier-reported strains were neither available nor correctly assigned phylogenetically. Thus, we investigated the metamorphosis-inducing capacity of 20 representative and phylogenetically characterized bacterial strains isolated from *Hydractinia*-covered shells (19–21), one phylogenetically related coral-associated strain (*Pseudoalteromonas* sp. strain PS5) (8), and eight bacterial type strains. All strains were tested in a previously reported monospecies biofilm-based metamorphosis assay (22), in which untreated larvae kept in artificial seawater served as the negative control and larvae treated with CsCl (final concentration of 6 mM) served as the positive control (15, 16). To evaluate the observations from the larva-based metamorphosis assay, four different states were defined and counted after 24 and 48 h (Fig. 1; also, see Fig. S1 at https://doi.org/10.5281/zenodo.4537693): (i) no induction, in which larvae continued swimming or underwent reversible attachment to the surface; (ii) settlement (SET), which included irreversible attachment to the surface (oral ending), contraction along the oral-aboral axis, and development of a flat disc with a small tip in the center, but not full elongation to a primary polyp; (iii) metamorphosis (MET), involving full transformation into a functional primary polyp, with visible formation of tentacles and development of a hypostome and stolons; and (iv) dead larvae with lysed body parts.

**FIG 2:**
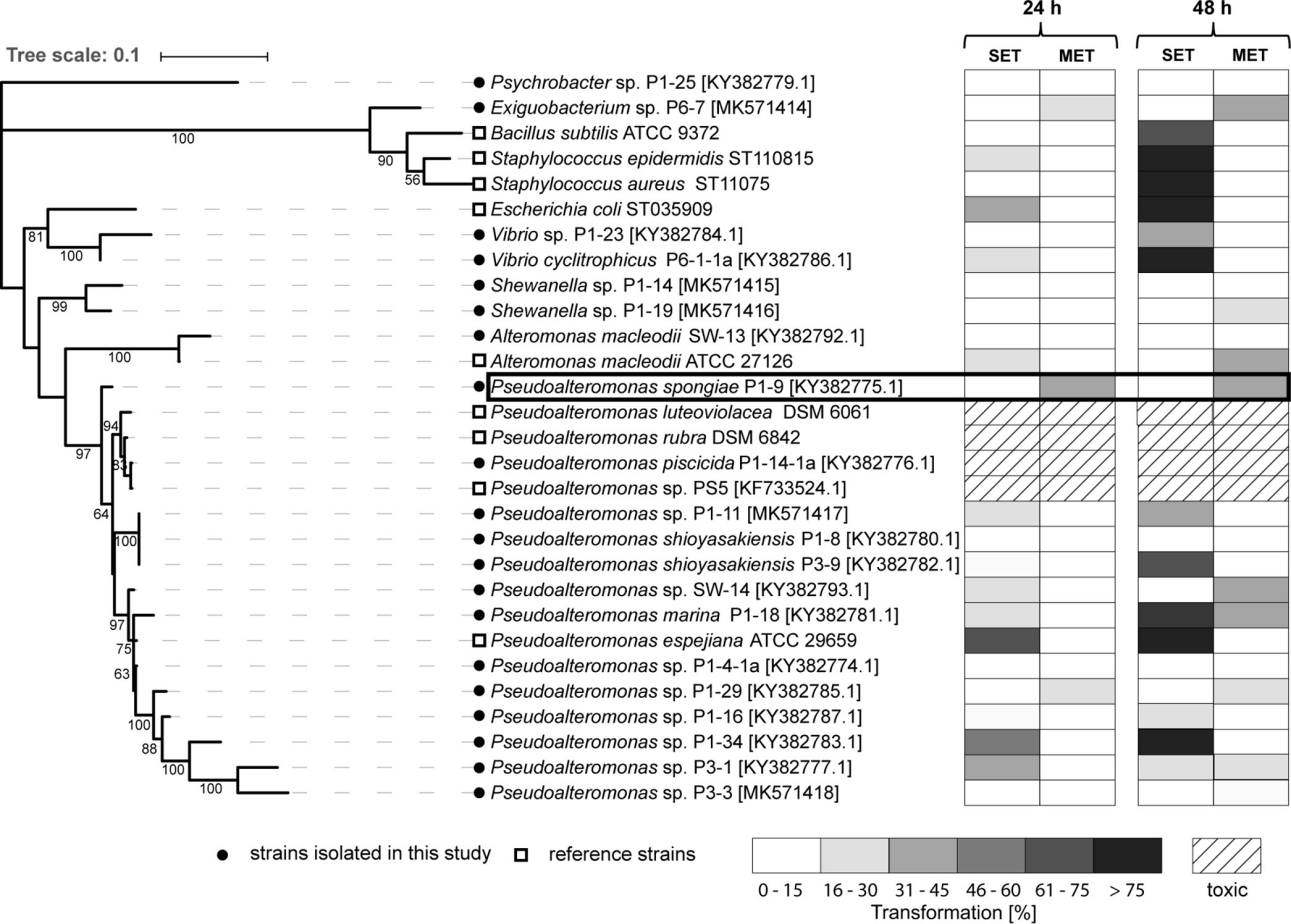
(Left) Phylogenetic tree based on 16S rRNA gene sequences of 29 tested bacterial strains. The best DNA model was generated, and the robustness of inferred tree topologies was evaluated after 1,000 bootstraps (>50% are shown). (Right) Heat map depicting the percent settled larvae (SET) and larvae that underwent metamorphosis (MET) after 24 and 48 h. Mean values of replicates are visualized as a color-coded 15% step gradient (*n* = 3; negative control, ASW; positive control, CsCl).

As shown in Fig. 2, three bacterial strains (*Pseudoalteromonas* sp. strain P1-9, *Pseudoalteromonas* strain P1-29, and *Exiguobacterium* sp. strain P6-7) were found to induce settlement and metamorphosis in the primary polyp within 24 h (see Fig. S3 at https://doi.org/10.5281/zenodo.4537693). An additional five strains (*Pseudoalteromonas* sp. strain P1-19, *Pseudoalteromonas* sp. strain SW-14, Pseudoalteromonas marina P1-18, *Pseudoalteromonas* sp. P3-1, and Alteromonas macleodii ATCC 27126) were found to induce settlement within the first 24 h, but subsequent formation of the primary polyp was observed only within 48 h. Up to 11 strains, including several Gram-positive reference strains, caused only settlement, but none of them induced the full transformation to the primary polyp, eventually causing the death of the transforming larvae. Six strains were found to be noninducing, and four strains, mostly belonging to the clade of pigmented *Pseudoalteromonas* strains (23), caused the death of up to 100% of all larvae within 24 h. Across all tested strains, *Pseudoalteromonas* sp. P1-9 was found to induce the most robust morphogenic response and thus was selected for further chemical analysis. In accordance with previous reports (15, 17, 18), it was also noted that *Hydractinia* responded in the presence of CsCl in a highly synchronized and morphologically distinct manner. It was found to tolerate the continuous presence of 6 mM CsCl without any signs of abnormal transformation. In contrast, nonsynchronized induction events in colony-based assays were observed, as reported earlier (18). This is presumed to have resulted from the complex nature of bacterial biofilms and the spatial fluctuation of the unidentified bacterial signaling molecules within the biofilm matrix.

### Bioassay-guided identification of bacterial signals from *Pseudoalteromonas* sp. P1-9.

**FIG 3:**
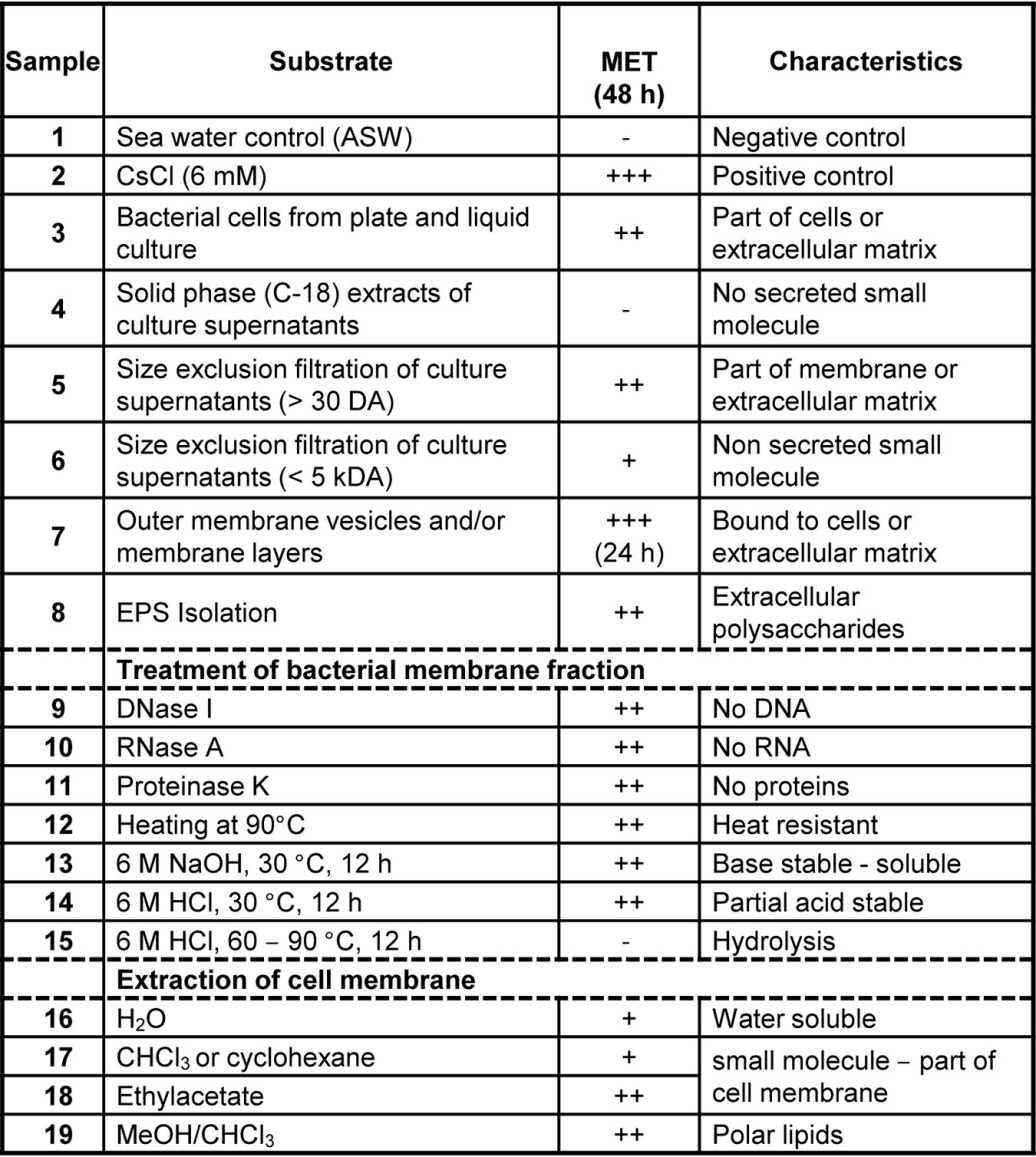
Metamorphosis assay of samples derived from Pseudoalteromonas spongiae P1-9. Percent metamorphosis was evaluated after 48 h (*n* = 3). For simplicity, activity was divided into four categories: no induction (–), <40% metamorphosis (+), 40 to 80% metamorphosis (++), and >80% metamorphosis (+++) (for details, see Fig. S3 to S10 at https://doi.org/10.5281/zenodo.4537693).

To test whether the morphogenic cue is a secreted, diffusible, small metabolite, solid-phase extracts (C_18_ cartridges) of cell-free culture supernatants were prepared (22). However, none of the metabolite extracts revealed morphogenic activities (sample 4). We then aimed to determine whether morphogens are secreted high-molecular-weight (HMW) components (e.g., proteins, cell surface-associated exopolysaccharides [EPS], or OMVs) or an integral part of cell membranes (Fig. 3; also, see Fig. S2 to S6 at https://doi.org/10.5281/zenodo.4537693). First, we analyzed if components of the cell membrane induce the transformation. To test this, bacterial cells (sample 3) were subjected to mild cell lysis and a series of centrifugation and washing steps. Then, the resulting concentrated cell membrane fraction was subjected to size exclusion separation. Subsequent bioactivity studies of separated fractions revealed that the HMW fraction (>30 kDa) induced up to 80% of all larvae to metamorphose in a dose-response manner (sample 5), while low-molecular-weight fractions (<5 kDa) caused metamorphosis in less than 40% of all larvae (sample 6).

**FIG 4:**
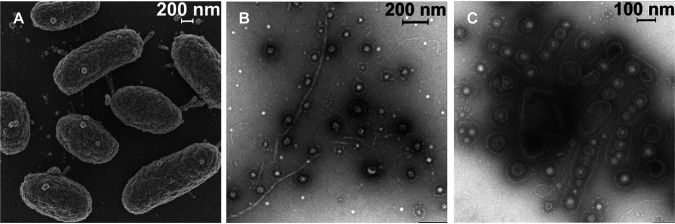
(A) Scanning electron microscopy of single cells of P1-9 obtained from a 3-day-old liquid culture. (B and C) Negative-contrast electron-microscopic image of vesicles coated with an S-layer-like matrix and string-shaped biopolymers isolated from a shaking P1-9 culture (marine broth, 3 days) (B) and a biofilm matrix derived from static growth (marine broth agar, 3 days) (C).

In a complementary approach, we also tested whether the morphogen is an integral part of OMVs and/or a soluble biopolymer (24–26). An OMV-enriched fraction was concentrated by ultracentrifugation of a sterile-filtered culture supernatant, which induced metamorphosis in more than 80% of all larvae within 24 h (sample 7). Surprisingly, an enriched exopolysaccharide fraction that was obtained by selective washing and centrifugation steps (sample 8 [see Fig. S10 at https://doi.org/10.5281/zenodo.4537693]) was found to have similar morphogenic activity. To confirm the presence of OMVs and/or biopolymers, the bacterial producer and OMV-rich samples were subjected to scanning electron microscopy (SEM) and cryo-transmission electron microscopy (cryo-TEM). As depicted in Fig. 4, obtained SEM images revealed that strain P1-9 actively releases OMVs, while cryo-TEM images of samples showed entire cells, minicells, and cells proliferating what appear to be outer membrane vesicles (100 to 300 nm) as well as a high abundances of extracellular EPS-like fibers (Fig. 4B and C).

At this stage, we hypothesized that at least two different types of bacterial cues (membrane components and exopolysaccharides) are responsible for morphogenic induction, both of which appeared to be an integral part of the outer cell membrane and/or of high molecular weight. This assumption was supported by the finding that both the aqueous and methanolic extracts of pelleted cells revealed morphogenic activity (samples 15, 17–19).

To determine the stability and chemical nature of the morphogens, bacterial cells were first lysed. Then, after washing and centrifugation, they were subjected to enzymatic and physical treatments prior to activity tests (see Fig. S5 to S9 at https://doi.org/10.5281/zenodo.4537693). As depicted in Fig. 3, activity was completely retained when the enriched HMW cell membrane fraction was treated with digestive enzymes such as DNase, RNase, or proteinase K and even when heated to 96°C for 10 min (entries 9 to 12). Treatment with aqueous 6 M NaOH or 6 M HCl (12 h, 30°C) partially solubilized the active morphogen (samples 13 and 14), and activity of both the soluble fraction and residue was mostly retained after neutralization. However, treatment with 6 M HCl at a higher temperatures (>60°C) gradually abolished the morphogenic activity of the sample. Taking these results together, we deduced that *Pseudoalteromonas* sp. P1-9 produces two different bacterial morphogens, which are chemically stable and likely associated with or derived from the bacterial outer membrane, and that the morphogen(s) is not a sensitive protein, nucleic acid, or unstable secreted metabolite.

### Analysis of morphogenic phospholipids.

**FIG 5:**
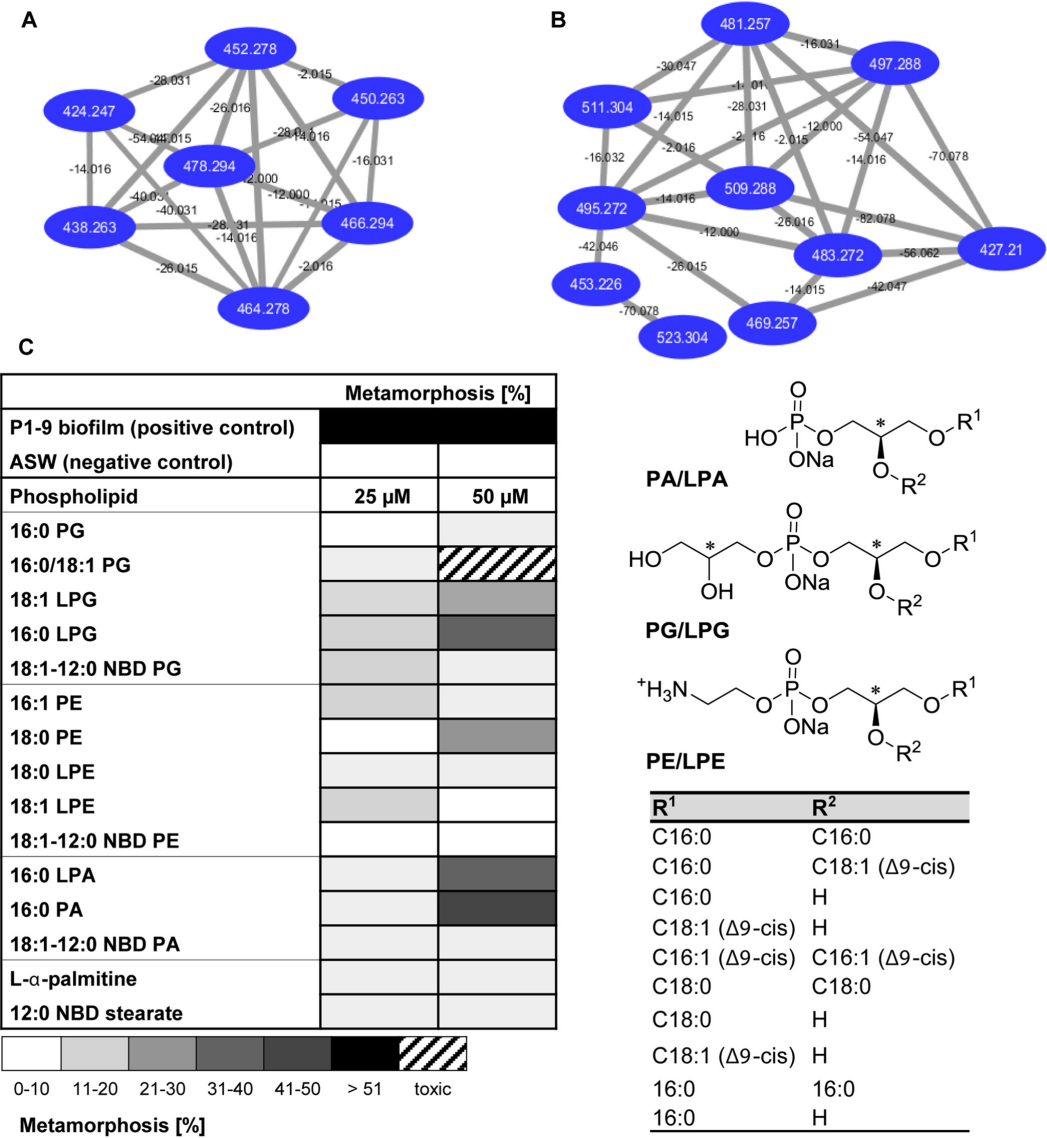
(A and B) LC–HR-MS/MS-based GNPS analyses of a purified lipid fraction showing the MS/MS-based cluster of LPE (A) and LPG (B). (C) Metamorphosis-inducing activity of commercial phospholipids. Test were performed at 25 and 50 μM, and transformation rates were determined after 24 and 48 h. Mean values for replicates are visualized as a color-coded 10% step gradient (*n* = 3; CsCl [positive control] is not shown).

In the next step, we focused on the purification and chemical analysis of methanolic extracts derived from washed and concentrated cell membranes (Fig. 3, sample 19), which reliably induced metamorphosis in *Hydractinia* larvae. Reverse-phase high-performance liquid chromatography (HPLC)-based purification of active fractions was followed by bioactivity assays, high-resolution tandem mass spectrometry (HR-MS/MS), and Global Natural Product Social Molecular Networking (GNPS) (27) analysis (Fig. 5A). In addition, morphogenic fractions were analyzed by ^1^H and ^13^C nuclear magnetic resonance (NMR). GNPS as well as NMR studies conclusively revealed the dominant abundance of phosphatidylglycerols (PGs), phosphatidylethanolamines (PEs), and their respective lyso- derivatives (LPGs and LPEs) (see Fig. S11 to S13 at https://doi.org/10.5281/zenodo.4537693) within the active fractions. Due to the inherent difficulties associated with the purification of closely structurally related phospholipids, 10 commercial derivatives with matching LC–HR-MS/MS patterns (see Fig. S40 to S50) and ^1^H and ^13^C NMR spectra (see Fig. S26 to S39) as well as two fluorescence-labeled derivatives were tested for their morphogenic activities in *Hydractinia* larvae (Fig. 5; also, see Fig. S14 to S15 and Table S3).

**FIG 6:**
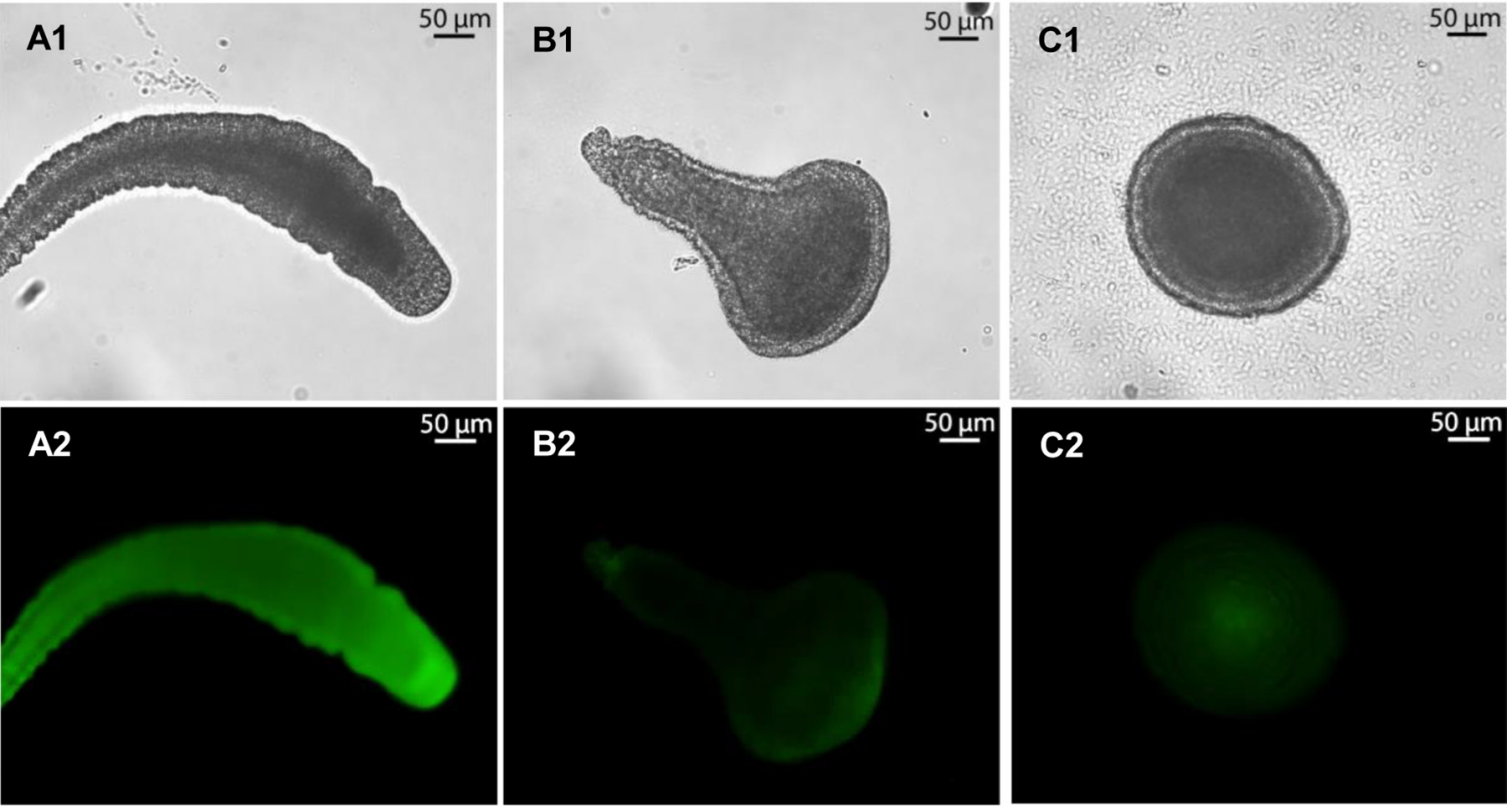
Metamorphosis assay with fluorescence-labeled phospholipids. Representative fluorescence labeling images of larvae treated with (A) 18:1 to 12:0 NBD PG after 5 h; (B) 18:1 to 12:0 NBD PE after 24 h; (C) 18:1 to 12:0 NBD PA after 24 h. (Top) Bright-field microscopy; (bottom) fluorescence imaging with an emission wavelength of 527 nm) (*n* = 3; the negative control [ASW] and positive control [CsCl] are not shown).

As displayed in Fig. 5, two lysophospholipids [1-palmitoyl-2-hydroxy-sn-glycero-3-phospho-(1'-rac-glycerol) (16:0 LPG) and 1-palmitoyl-2-hydroxy-sn-glycero-3-phosphate (16:0 LPA)], 1,2-distearoyl-sn-glycero-3-phosphoethanolamine (18:0 PE), and 1,2-dipalmitoyl-sn-glycero-3-phosphate (16:0 PA) repeatedly induced settlement and metamorphosis in 20 to 40% of all larvae within 48 h, while phospholipid concentrations exceeding 50 μM occasionally caused lysis of larvae (Fig. 5). It was also noted that some derivatives induced settlement of larvae but were unable to induce full metamorphosis, causing the eventual death of the transforming organism. We investigated whether a low solubility of (lyso)phospholipids could prevent the uptake and perception by *Hydractinia* larvae. Thus, in a following step, competent larvae (∼30 larvae) were exposed to artificial seawater (ASW) containing nitrobenzoxadiazole (NBD)-labeled phospholipids (18:1 to 12:0 NBD-PG, 18:1 to 12:0 NBD-PE, or 12-NBD stearate [Fig. 6; also, see Table S3 at https://doi.org/10.5281/zenodo.4537693]). After treatment, larvae were kept on glass slides covered with artificial seawater, and fluorescence was monitored over a period of 24 h. As depicted in Fig. 6, NBD-labeled (phospho)lipids were incorporated into the larvae tissue (80% of all tested larvae showed NBD-based fluorescence within a few hours), some of which metamorphosed into the primary polyp within 24 to 48 h (<20% of all larvae). During metamorphosis, the fluorescence signal continuously decreased, presumably due to the internalization and cellular decomposition of phospholipids (see Table S3). It was deduced at this stage that phospholipids are readily taken up and disseminated within the larval tissue and thus may be able to enter and interfere with intercellular signaling pathways.

**FIG 7:**
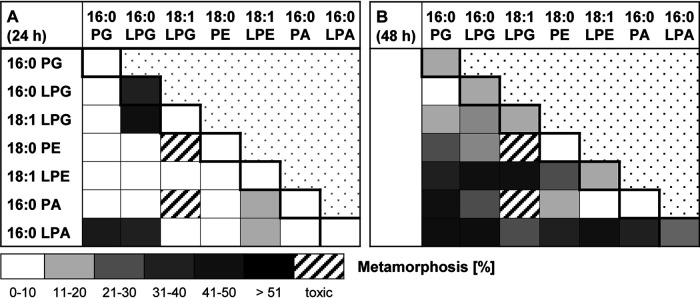
Metamorphosis assay of phospholipid combinations (a 25 μM concentration of each lipid in a 1:1 combination). Transformation rates were monitored after (A) 24 h and (B) 48 h. Mean values for replicates are visualized as a color-coded 10% step gradient (*n* = 3; the negative control [ASW] and positive control [CsCl] are not shown).

As phospholipid-enriched extracts are composed of a diverse set of (lyso)phospholipids and reliably induced morphogenesis, we investigated whether phospholipids in combination would show synergistic activities. As a proof of principle, larvae were exposed to 1:1 combinations of two lipids with only weak morphogenic activity (each at 25 μM). Several combinations (18:1 LPE–16:0 LPG, 18:1 LPE–18:1 LPG, 16:0 PA–16:0 PG, 16:0 LPA–16:0 LPG, and 16:0 LPA–18:1 LPE) revealed additive, if not synergistic, morphogenic activity, inducing up to 50% of all larvae to metamorphose within 48 h (Fig. 7), while other combinations appeared to have toxic effects (28).

**FIG 8:**
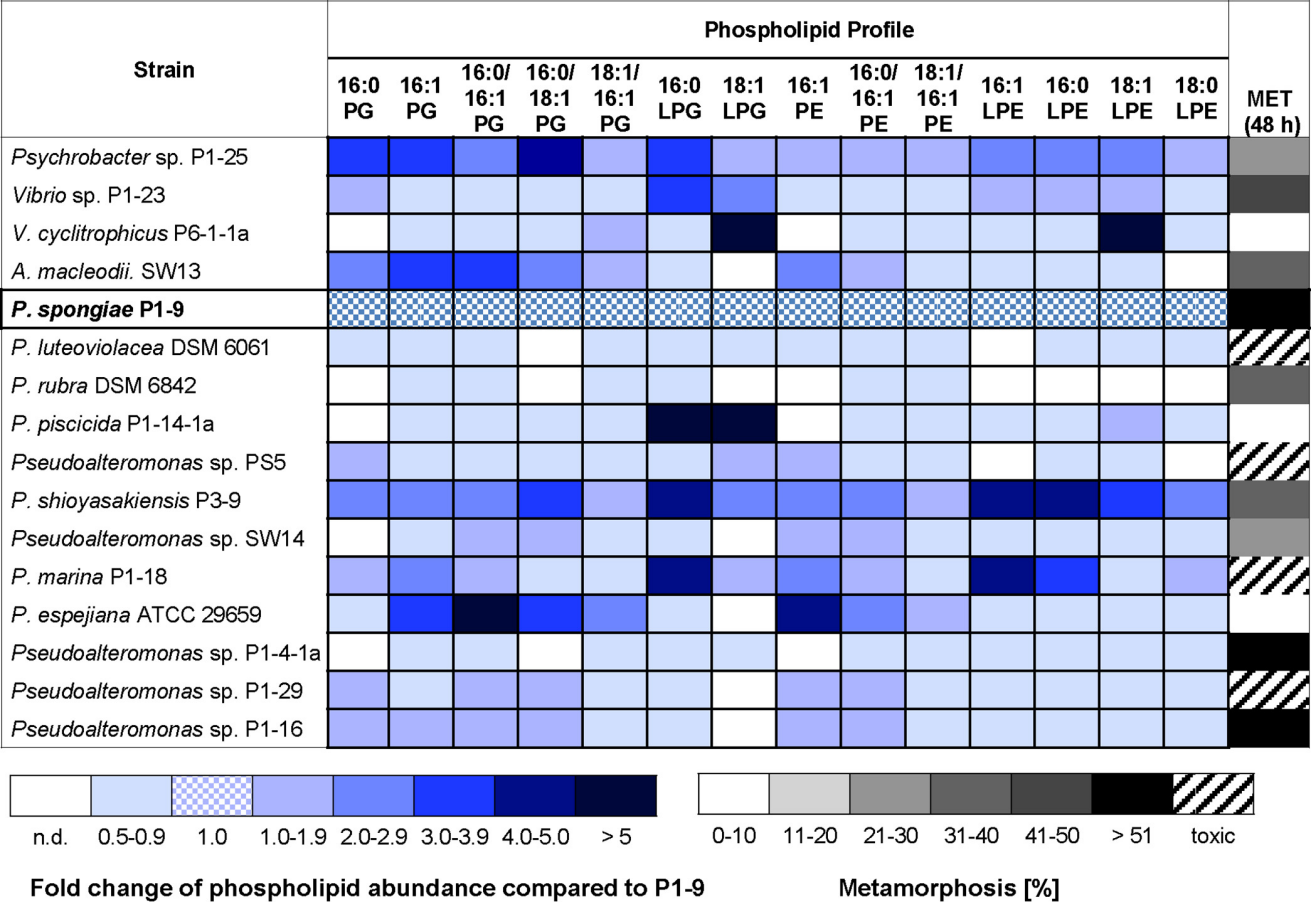
Relative phospholipid analysis of enriched cell extracts from different bacterial isolates (phospholipid abundance in Pseudoalteromonas spongiae P1-9 was set at 1.0) and metamorphosis assay with bacterial lipid extracts (100 μg/ml). Transformation rates were evaluated after 48 h. Mean values of replicates are visualized as a color-coded 10% step gradient (*n* = 3; the negative control [ASW] and positive control [CsCl] are not shown).

Next, we investigated whether other morphogenic bacteria produce morphogenic (lyso)phospholipids and whether the natural composition stimulates morphogenesis (Fig. 8). Lipid extracts obtained from six bacterial strains (*Vibrio* sp. strain P1-23, *A. macleodii.* SW13, Pseudoalteromonas rubra, Pseudoalteromonas shioyasakiensis P3-9, *Pseudoalteromonas* sp. strain P1-4-1a, and *Pseudoalteromonas* sp. strain P1-16) were found to induce metamorphosis to the primary polyp within 48 h, while lipid extracts of Vibrio cyclitrophicus P6-1-1a, Pseudoalteromonas piscicida P1-14-1a, and *P. espejiana* ATCC 29659 revealed no inducing activity. The extracts of four other strains caused cell lysis and/or larva death. These results were intriguing, as they revealed that bacterial species that were previously anticipated as being noninducing within the biofilm assay (e.g., *Psychrobacte*r sp. strain P1-25 and *Vibrio* sp. strain P1-23) produce morphogenic phospholipid combinations, while others that contained phospholipids similar to that of P1-9 were unable to induce morphogenesis. Thus, we concluded at this stage that the species-specific phospholipid composition plays a crucial role in triggering and/or preventing the morphogenic transition and that other, yet-unidentified metabolites and lipids might have inhibitory activity and thus counteract the inductive effects (29). Along these lines, we could verify the presence of known antibiofouling metabolites (violacein and/or polybrominated pyrrole derivatives) in the lipid extracts that were toxic to larvae (*Pseudoalteromonas* sp. strain PS5 and Pseudoalteromonas luteoviolacea) (see Fig. S51 to 55 at https://doi.org/10.5281/zenodo.4537693).

### Analysis of morphogenic polysaccharides.

**FIG 9:**
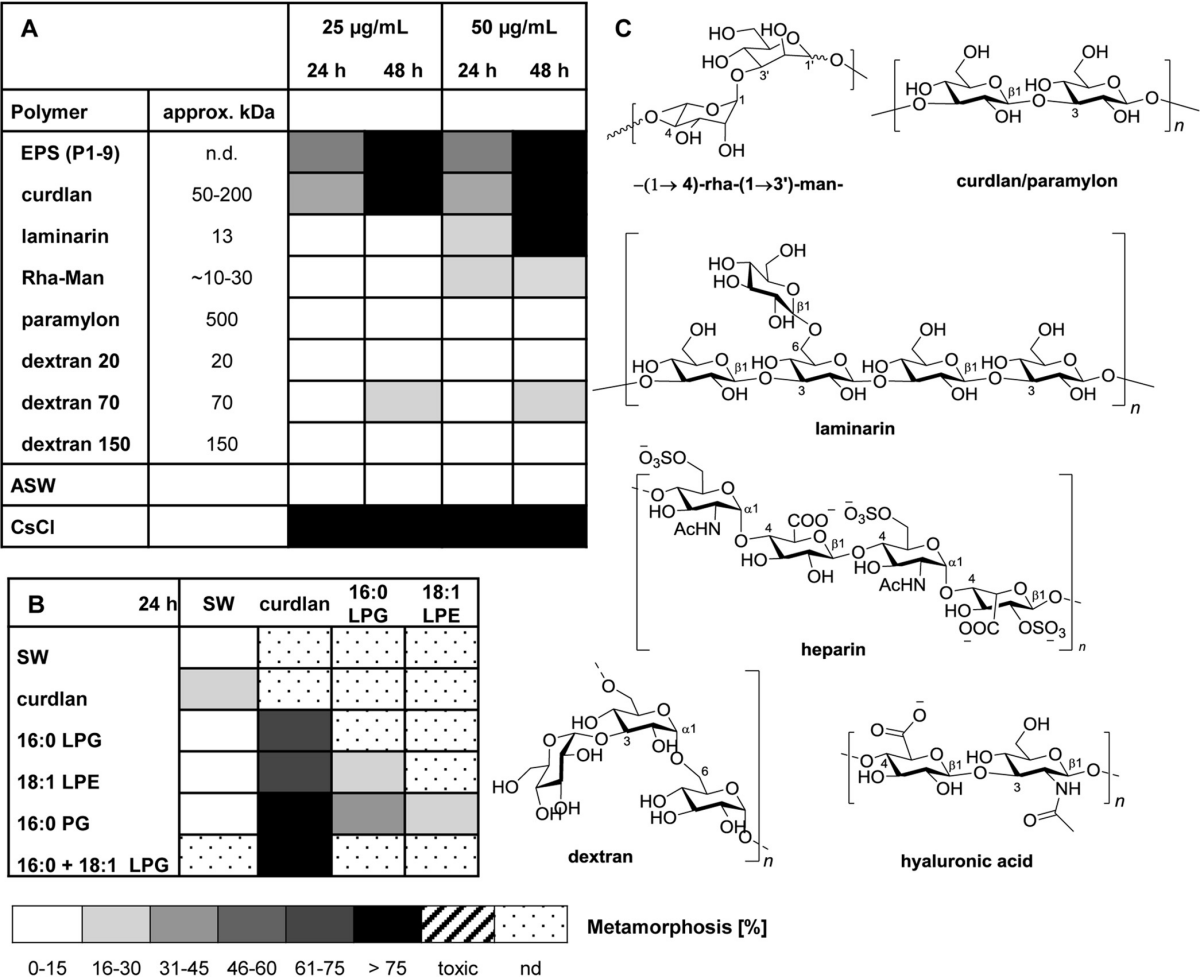
(A) Metamorphosis assay with enriched and purified polysaccharides from P1-9 and commercial polysaccharides (25 μg/ml) (*n* = 4); transformation rates were determined after 24 h and 48 h. (B) Metamorphosis assay with commercial phospholipids (10 μg per lipid) and curdlan (15 μg) after 24 h. Mean values of replicates are visualized as a color-coded 15% step gradient (*n* = 3). (C) Representative structures of repeating units of tested polysaccharides. n.d., not defined.

Having identified the lipid-based morphogenic factors, we then focused on the analysis of the most active HMW fractions (metamorphosis of up to 80 to 100%) retrieved from aqueous extracts of bacterial biomass (Fig. 2). After size exclusion filtration of aqueous extracts, fractions that caused more than 20% of all larvae to metamorphose were analyzed by mass spectrometry (HR-MS and matrix-assisted laser desorption ionization [MALDI]) and ^1^H NMR, which indicated the presence of a complex mixture of unknown polysaccharides (see Fig. S9 and S21 at https://doi.org/10.5281/zenodo.4537693). Subsequent bioassay-guided size exclusion purification by Sephadex G25, followed by NMR and HR-MS/MS analysis of the most active fraction, resulted in the identification of a morphogenic polysaccharide consisting of repeating (1′→4)-α-l-Rha-(1→3′)-d-Man-units (see Table S2 and Fig. S19 to S25). The rhamnose/mannose composition was confirmed by acid hydrolysis using 6 M HCl, followed by trimethylsilyl (TMS) derivatization, gas chromatography-mass spectrometry (GC-MS) analysis, and comparison with commercial standards (see Fig. S16). The purified polysaccharide, referred to here as Rha-Man, showed a clear dose-dependent morphogenic induction of up to 80% within 48 h (150 μg/ml) (see Fig. S17). Partial acid hydrolysis resulted in the loss of morphogenic activity; similarly, rhamnose, mannose, and glucose monomers displayed no inducing effect. Here, it needs to be noted that comparative NMR analysis of bioactive HMW fractions clearly indicated that Rha-Man was not the only EPS within the bioactive fraction (see Fig. S19), which suggested that other, structurally related EPS derivatives might also contribute to the morphogenic activity of the EPS fraction. Due to the inherent difficulties associated with the separation and structural characterization of polysaccharides, we subsequently tested commercial and structurally defined bacterial polysaccharides (Fig. 9). Interestingly, curdlan, which is a well-known polysaccharide (50 to 200 kDa) with a β-1,3-glycosidic linkage that is produced by the Gram-negative bacterium Alcaligenes faecalis, induced more than 50% of all larvae to undergo full metamorphosis within 48 h. In contrast, paramylon, the 500-kDa derivative of curdlan, which is distinct in its average length and three-dimensional structure (30), and charged EPS derivatives, such as hyaluronic acid and heparin, did not induce metamorphosis.

Due to the natural co-occurrence of EPS and lipopolysaccharide (LPS) within bacterial biofilms, we questioned whether combinations would exhibit the morphogenic activities observed for enriched OMVs or biofilms. Indeed, combinations of curdlan (15 μg/ml each), with 16:0 LPG, 18:1 LPE (10 μg/ml each), or both, induced metamorphosis in more than 75% of all larvae, causing the formation of a fully functional primary polyp within 24 h (Fig. 9B).

## DISCUSSION

The broadly distributed marine hydrozoan *Hydractinia echinata* has become a model organism for metamorphosis of marine invertebrates. Artificial inducers, most notably CsCl, have long served as reliable tools to artificially induce metamorphosis in a synchronized fashion; however, the identity of the natural bacterial signals and the mechanisms of the underlying bacterium-host interaction remained unknown.

Here, we report the identification and evaluation of two types of bacterial metabolites, a subset of (lyso)phospholipids and exopolysaccharides, that alone and in combination induced the morphogenic transformation of *Hydractinia* planula larvae into the primary polyp. Most notably, bacterial lysophospholipids (16:0/18:1 LPG, 18:0 LPE/PE, and 16:0 LPA/PA) induced metamorphosis as single compounds and in combination with other phospholipids, while other phospholipids and combinations did not trigger any transformations. These findings are also in agreement with the morphogenic activity of enriched OMVs fractions derived from P1-9. Although there is no consensus on a conserved mechanism of OMV release, they are known to contain natural components of the bacterial outer membrane, including phospholipids (31–33). Our study also demonstrated that other phospholipid mixtures derived from different bacterial lineages are able to initiate metamorphosis. In a complementary approach, we also identified that bacterial exopolysaccharides, e.g., a Rha-Man-based polysaccharide from *Pseudoalteromonas* sp. P1-9 and curdlan derived from *A. faecalis*, induce metamorphosis in a dose-response fashion. While the presence of one morphogenic signal type is sufficient to induce morphogenesis in a subset of larvae, combinations of phospholipids and curdlan induced polyp formation at rates that exceeded in part the sum of single compound contributions, resulting in the complete transformation of almost all tested larvae within 24 h.

Both bacterial (lyso)phospholipids (31–33) and exopolysaccharides (34–38) are highly structurally diverse compound classes and are known to co-occur in bacterial biofilms, as they are integral components of cell membranes and biofilms. However, their abundances are not only species specific but also strongly dependent on the metabolic state of the producing bacteria (39). Considering the natural interaction of bacterial biofilms and habitat-exploring *Hydractinia* larvae, it is reasonable to assume that larvae are likely to encounter both morphogens in various compositions and concentrations while actively searching benthic biofilm-covered surfaces. Larvae are thus required to quickly distinguish between beneficial and more repellent environments before initiating the irreversible transformation.

In the case of phospholipids, our results suggest that the physical contact of larvae with lipid-rich vesicles and cell fragments naturally present within marine biofilms should allow the detection of lipids, while the lipid and exopolysaccharide composition are decisive for initiating morphogenesis. For *Hydractinia* larvae, it was proposed early on that neurosensory cells, dominantly located at or near the anterior pole and in part on the tapered posterior tip, are responsible for the detection of the bacterial cues (16, 17). As LPAs have been recognized as potent mitogens in humans due to their interactions with G-protein-coupled receptors (GPCRs), thereby altering many different cellular responses, such as proliferation, survival, cytoskeletal changes, and calcium influx (40), a homologous mode of action could be hypothesized for *Hydractinia*. Furthermore, the passive integration of lipids into larval tissue could induce changes in membrane fluidity, which indirectly results in the recruitment of, for example, protein kinase C (PKC), which is involved in cellular signaling processes (41–45). In contrast, polysaccharides require detection via dedicated receptors, as described for EPS-mediated host-pathogen interactions (46) and the metamorphosis of other marine animals (47). In particular, curdlan was previously reported to act on lectin-type receptors in humans; this receptor type was also detected in a previous *Hydractinia* transcriptome study (48).

Our findings are intriguing, as synergistic effects of morphogens, often of lipid and polysaccharide origin, were recently reported in other marine model systems. A closely related observation was reported for coral larvae, which transformed upon exposure to crustose coralline algae, and glycoglycerolipids and a polysaccharide were identified as settlement cues (49). In another study, combinations of bacterial sulfonolipids (also termed rosette-inducing factors, or RIFs) and LPEs were reported to induce the formation of a predatory rosette-like stage in the choanoflagellate Salpingoeca rosetta (50–52), while synergistically acting nucleobases from marine bacteria were found to induce the metamorphosis of the invasive fouling mussel Mytilopsis sallei (53).

### Conclusion.

In this study, we identified two types of morphogenic cues from marine bacteria naturally associated with *H. echinata* that cause larvae to settle and metamorphose. The bacterial metabolites identified satisfy the ecological criteria for acceptance as an inductive cue, due to their abundance in bacterial biofilms, OMVs, and cell membranes. Our findings show similarities to other cnidarian settlement cues and thus raise the question of whether complex lipids and polysaccharides are general metamorphic cues for cnidarian larvae. Our results allowed us to propose *Hydractinia* as a model system to more closely investigate host-microbe interactions, to shed light onto the long-standing mystery of how bacterial signals trigger animal development in the marine world, and to open new avenues for future studies on their target receptors.

## MATERIALS AND METHODS

### Cultivation of *Pseudoalteromonas* sp. P1-9.

A preculture of marine bacteria was grown for 3 days at 30°C (160 rpm) in marine broth (MB) medium and used as the inoculum. For isolation of inducing signals, a 7-ml preculture of *Pseudoalteromonas* sp. P1-9 was used to inoculate 500 ml MB and incubated at 30°C for 3 days (150 rpm). Then, cultures were centrifuged for 20 min at 4°C (4,000 rpm), and the supernatant was separated from the cell pellet. The cell pellet was washed three times (suspended in 5 ml sterile phosphate-buffered saline [PBS] and centrifuged [20 min, 4°C, 4,000 rpm]; the supernatant was discarded) and then suspended in 1 ml sterile water and sonicated on ice (5 time for 30 s with a 30-s break). To remove soluble cytosolic components, samples were centrifuged (10,000 rpm, 10 min, 4°C), the supernatant was discarded, and the resulting cell pellet was lyophilized overnight (P1-9A samples). The lyophilized cell pellet was used for further enzymatic (DNase and RNase) and chemical treatments (see the supplemental information at https://doi.org/10.5281/zenodo.4537693 for details).

### *H. echinata* husbandry.

*H. echinata* colonies were obtained as single colonies on gastropod shells from the Alfred Wegener Institute, Helmholtz Centre for Polar and Marine Research (Helgoland, Germany). Adult polyps on shells were kept in artificial seawater (salinity of 33.2 to 33.7‰, pH 8.2 to 8.3, and 16°C) in aerated tanks maintained with a 16-h light/8-h dark cycle and were fed daily using 3- to 7-day-old nauplii of Artemia salina. Fertilized eggs were collected in the two- to four-cell stage 3 h after the spawning event and transferred into freshly sterile seawater.

### Bioassay.

After the spawning event, fertilized eggs were collected and kept in sterilely filtered artificial seawater (ASW) at 18°C. After 2 days, developing larvae were washed two or three times with sterile ASW and transferred to a new sterile petri dish filled with fresh ASW. The procedure was repeated every second day until use, and larvae were kept at 20°C. For bioassays, 20 to 30 competent larvae (kept in ASW) were added to a test well (sterile 24-well plates), and the wells were filled with ASW to reach 2 ml. For all experiments, negative controls (larvae kept in sterile ASW) and positive controls (larvae induced with a CsCl solution [6 mM final concentration]) were included. Experiments were performed at 20°C in triplicate and repeated with larvae from at least two different spawning events.

### Colony-based assay.

Bacterial strains were grown on a marine broth (MB) agar (Carl Roth) or LB agar for 3 days. A single colony was suspended in 100 μl PBS (optical density at 600 nm [OD_600_] of 0.2), and 5 μl of the bacterial suspension was transferred to a sterile well (24-well plates). Cells were incubated for 20 min under a laminar flow to allow surface attachment and then used for testing.

### Testing of biosamples and chemical extracts.

Samples were centrifuged at 10,000 rpm for 10 min at 4°C, resulting in an insoluble pellet and supernatant. For assays, the pellet was suspended in 1 ml sterile seawater, and supernatant was used without further dilution. For testing, 60 μl of each sample was added to a sterile 24-well plate, dried under a sterile laminar flow for 20 min, and then used for testing.

### Testing of phospholipids.

Each (lyso)phospholipid was dissolved in methanol (MeOH) to give a 1-mg/ml stock solution. The respective test amount was added to each well of a sterile well plate (e.g., sterile 24-well plates). Samples were dried under a sterile laminar flow and then used for testing.

### Testing of commercial polysaccharides.

Exopolysaccharides were dissolved in double-distilled water (ddH_2_O) to give a 1.0-mg/ml stock solution and sterilized by membrane filtration (0.22 μm). Insoluble polysaccharides, e.g., curdlan and paramylon, were autoclaved at 120°C for 10 min. For assays, 100 μl or 50 μl of each stock solution was applied per well, dried under a sterile laminar flow, and then used for testing.

### Structure elucidation. (i) Analysis of phospholipid content.

Bacteria were cultured in 50 ml MB (180 rpm, 30°C, 2 days), and supernatant was separated by centrifugation (4,000 × *g*, room temperature, 20 min) and discharged. The cell pellet was collected, lyophilized, and extracted twice with MeOH (10 ml) assisted by ultrasonication. Methanolic extracts were dried and redissolved in MeOH to yield a 5-mg/ml stock solution for testing. For LC-MS analysis, the stock solution was diluted to yield a 0.1 m/ml solution for measurements.

### (ii) HPLC–HR-MS analysis.

Measurements were performed on a Thermo Q-Exactive Plus mass spectrometer using the following gradient: 0 to 1 min, 20% B; 1 to 2 min, 20% to 40% B; 2 to 25 min, 40% to 92.5% B; 25 to 26 min, 92.5% to 100% B; 26 to 35 min, 100% B; 35 to 35.1 min, 100% to 20% B; 35.1 to 38 min, 20% B (A, ddH_2_O with 20 mM ammonium formate [pH 4.5]; B, 50% isopropyl alcohol [*i*PrOH]–50% acetonitrile [MeCN]). The flow rate was 0.3 ml/min. Metabolite separation was followed by a data-dependent MS/MS analysis in both positive (MS^1^) and negative (MS^1^ and MS^2^) ionization modes.

### (iii) GNPS-mediated molecule networking.

Metabolomic raw data files were recorded on Thermo QExactive MS instrument and were first converted to .mzXML by ProteoWizard and then uploaded to the GNPS (Global Natural Products Social Molecular Networking) server.

### (iv) Purification of phospholipids.

The lyophilized cell pellet derived from 1 liter of culture (MB; 30°C, 2 days, 120 rpm) was extracted with 500 ml MeOH. Insoluble cell debris was filtered off, and the resulting methanolic filtrate was collected and concentrated under reduced pressure. The extract was loaded on a silanol (SiOH) solid-phase extraction (SPE) cartridge (2 g), and metabolites were eluted using a step gradient of solvent mixtures (cyclohexane, ethyl acetate [EtOAc], and MeOH). Metabolites were collected in eight fractions, and the solvent was removed under reduced pressure. The metabolite content of each fraction was analyzed by ultra-high-performance liquid chromatography-mass spectrometry (UHPLC-MS) and NMR, and the morphogenic properties were evaluated.

### (v) Purification of polysaccharide.

Lyophilized cell pellet was extracted with ddH_2_O, and the resulting crude extract (170 mg) was purified by size exclusion chromatography in Sephadex G25 medium. The active fraction (orange band [53 mg]) was again lyophilized, suspended in 1 ml H_2_O, heated to 95°C for 1 h, then cooled on ice for 5 min, and subjected to DNase, RNase, and proteinase K treatments. The resulting solution was centrifuged (13,000 rpm, 20 min), filtered through 0.45-μm filters, and subjected to semipreparative size exclusion chromatography (Shodex GF310HQ column) using a 20 mM aqueous ammonium acetate (pH 9) solution for elution. Collected fractions were lyophilized and subjected to the metamorphosis assay and NMR analysis.

### (vi) SEM.

Bacteria were fixed with glutaraldehyde (2.5% [vol/vol]) for 1 h at room temperature while sedimenting on poly-l-lysine-coated coverslips. After two washings in cacodylate buffer (100 mM, pH 7.2), cells were dehydrated by increasing concentrations of ethanol, followed by critical-point drying in a Leica EM CPD300 automated critical-point dryer (Leica, Germany). Then, the coverslips were mounted on aluminum sample holders (stubs) and gold sputter coated (layer thickness, 20 nm) in a BAL-TEC SCD 005 sputter coater (BAL-TEC, Liechtenstein). Images were acquired with a Zeiss (LEO) 1530 Gemini field emission scanning electron microscope (Zeiss AG, Germany) at a 4-kV acceleration voltage and a working distance of 3 to 4 mm using an Inlense secondary electron detector.

### (vii) Cryo-TEM.

A drop of a freshly prepared OMV solution (2 μl) was placed on a R3.5/1 holey carbon-coated copper grid (Quantifoil Micro Tools GmbH, Germany). The sample was rapidly plunge-frozen in liquid ethane at −180°C. The frozen grid was transferred into a liquid nitrogen-cooled Gatan 626-DH cryo-holder (Gatan Inc., USA) and inserted into a Philips CM 120 cryo-TEM (Philips, Netherlands) operated at a 120-kV accelerating voltage. Images were acquired with a 1k × 1k FastScan-F114 charge-coupled device (CCD) camera (TVIPS GmbH, Germany). An isolated OMV/minicell solution (20 μl) was placed for 1 min onto hydrophilic, Formvar/carbon-coated copper grids (Quantifoil Micro Tools GmbH, Germany), washed twice on drops of distilled water, and stained on a drop of 2% uranyl acetate in distilled water. Samples were imaged in a Zeiss EM902A electron microscope (Carl Zeiss AG, Germany) operated at an 80-kV accelerating voltage. Images were acquired with a 1k × 1k FastScan-F114 CCD camera (TVIPS GmbH, Germany).

### Data availability.

Supporting information can be accessed at https://doi.org/10.5281/zenodo.4537693 and contains data on cultivation, bioassays, and metabolic analysis and structural characterization of bacterial morphogens.
